# Development and psychometric evaluation of self‐management scale for pregnant woman with gestational diabetes mellitus in China

**DOI:** 10.1002/nop2.1202

**Published:** 2022-02-27

**Authors:** Guofang Kuang, Xin Meng, Yiqian Wang, Ru Xu, Meng Zhang

**Affiliations:** ^1^ 235960 Department of Obstetrics The Affiliated Hospital of Qingdao University Qingdao China

**Keywords:** gestational diabetes mellitus, obstetrical nursing, pregnant woman, reliability, self‐management scale, validity

## Abstract

**Aim:**

To develop a self‐management scale and evaluate its validity for pregnant woman with GDM in China.

**Design:**

A cross‐sectional survey design.

**Methods:**

This study was conducted through three phases. The item pools of the scale were developed through literature review and expert interview. Content validity was assessed by an expert panel. Structure validity was evaluated through exploratory factor analysis. In the end, internal consistency reliability was tested.

**Results:**

The self‐management scale includes four dimensions, including self‐management consciousness, pregnancy management, blood glucose management and resource utilization, with a total of 35 items. In the scale, the Cronbach's α was 0.95. The split‐half reliability of the overall scale is 0.79. And the test‐retest reliability was 0.91. The content validity was 0.94.

**Conclusions:**

The scale is significantly valid and reliable, and it can be used to evaluate the self‐management ability of pregnant woman with GDM in China.

## 1 INTRODUCTION

Diabetes mellitus, a non‐communicable chronic disease, has become a major challenging health problem all over the world (Kamradt et al., 2014; Veeraswamy et al., 2012). It was reported by the International Diabetes Federation (IDF Diabetes Atlas) that 463 million adults suffered from diabetes in 2019 and 9.3% of adults aged 20–79 years are living with diabetes. IDF estimates that there will be 578 million adults with diabetes by 2030, and 700 million by 2045. Diabetes is one of the fastest growing health challenges of the 21st century, with the number of adults living with diabetes having more than tripled over the past 20 years (2019). Particularly, gestational diabetes mellitus (GDM) is one of the most common complications of pregnancy. Gestational Diabetes Mellitus refers to any degree of glucose intolerance with onset or first recognition during pregnancy. GDM currently affects approximately 2%–10% of pregnant women in the United States, 2%–6% in Europe, 5%–8% in Australia and 10%–15% in China (Carolan‐Olah, 2016). Chinese women, compared to women of many other ethnic backgrounds, are at higher risk of developing GDM. Women with GDM are at high risk of serious health outcomes such as hypoglycaemia and respiratory difficulties, macrosomia or high infant weight, premature delivery, birth damage, labour dystocia, even hypertension and heart disease in later than mother without GDM though GDM generally resolves once the baby is born. The offspring of mothers with GDM are predisposed to childhood obesity, early onset of type 2 diabetes and cardiovascular disease in adult life (Mensah et al., 2020; Veeraswamy et al., 2012). Therefore, it is urgent to control blood glucose level and protect the health of the mother and the foetus once GDM is diagnosed.

## 2 BACKGROUND

Self‐management scale is an effective tool for pregnant women with GDM to prevent these complications and achieve optimal blood glucose level during pregnancy. Generally, self‐management scale includes self‐monitoring blood glucose, dietary modification and increasing physical exercise (Mensah et al., 2020). Self‐management is the individual's ability to manage the symptoms, treatment, physical and psychosocial consequences and life style changes inherent in living with a chronic condition (Barlow et al., 2002). Self‐management skill is necessary to enable pregnant women managing their own GDM. Therefore, a valid and reliable tool, which assesses self‐management behaviour in women with GDM, is needed. The Summary of Diabetes Self‐Care Activities measure (SDSCA) is one of the most popular and frequently used tools in English‐speaking regions. The questionnaire is an 11‐item self‐reporting tool assessing levels of self‐care in adults with diabetes (Toobert et al., 2000). The Diabetes Management Self‐Efficacy Scale (DMSES) is another widely used scale and also some countries such as Australia, United Kingdom and China had accepted the use of the scale as a best practiced model (Sturt et al., 2010). In addition, educational and intervention programs, such as a Web‐based intervention, are also useful to improve knowledge of GDM and GDM self‐management principles (Carolan‐Olah et al., 2015; Liu et al., 2021; Wan et al., 2019).

Generally, the self‐management scale is used for patients with general chronic diabetes, not for pregnant woman with gestational diabetes mellitus (Gharaibeh et al., 2017; Schmitt et al., 2013; Sousa et al., 2010). Rossella Messina developed an Italian version of the diabetes management self‐efficacy scale for type 2 diabetes (Messina et al., 2018). Martina Kamradt constructed a German version of the Summary of Diabetes Self‐Care Activities measure and assessed self‐management in patients with diabetes mellitus type 2 in Germany (Kamradt et al., 2014). Wah et al. (2018) explored self‐management of gestational diabetes among Chinese migrants in Australia. Currently, there is lack of self‐management scale for pregnant woman with gestational diabetes mellitus in China. Therefore, it is urgent to develop a self‐management scale for pregnant woman with gestational diabetes mellitus in China and testify its psychometric validity, provide an assessment tool for developing effective nursing intervention measures, and improve pregnant women's self‐management ability.

## 3 METHODS

### 3.1 Design

This cross‐sectional survey study was conducted through three phrases to develop and evaluate psychometrical properties of self‐management scale for pregnant women with GDM. In Phase 1, the item pools of the scale were developed through literature review and expert interview with 9 experts from our hospital. In Phase 2, a Delphi survey was conducted to evaluate the authority and coordination among 28 experts from 20 hospitals in different cities of China. In Phase 3, the validity and reliability of the scale were tested among 190 participants of the pregnant women with GDM in our hospital.

#### 3.1.1 Development of the initial self‐management scale

**FIGURE 1:**
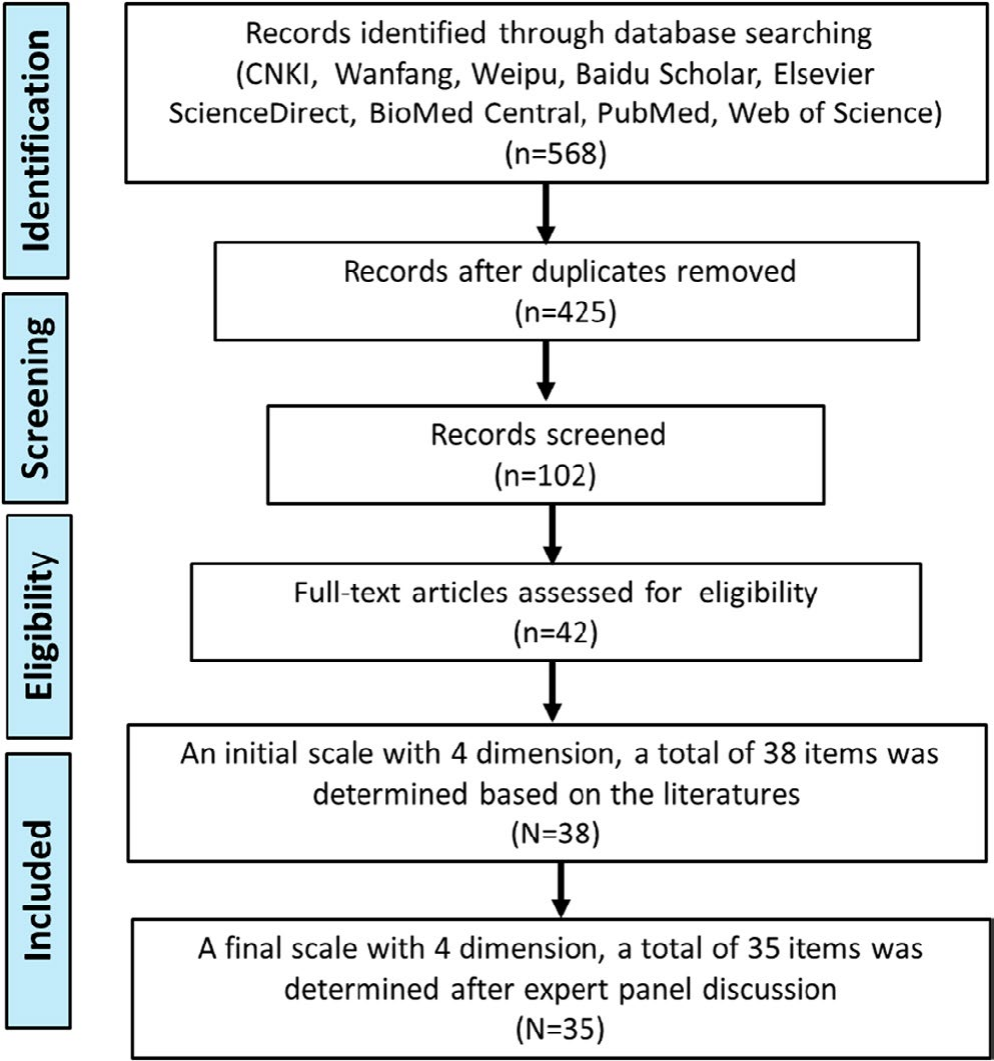
Prisma flow diagram for searching literature and identifying initial items of self‐management scale

A range of literatures were searched using Chinese and international databases, including Web of Science, Elsevier Science Direct, BioMed Central, PubMed, Scopus, CNKI (China National Knowledge Infrastructure), Wanfang, Weipu and Baidu. The published time range is limited from 2010 to 2020. The key words or topic phrases were chosen as pregnant women, diabetes, gestational diabetes mellitus (GDM), blood glucose control, dietary modification, physical exercise, weight management in pregnancy, and GDM lifestyle intervention. Papers published in English and Chinese are eligible for inclusion. As a result, 568 papers were chosen based on the database searching. After removing duplicates, 102 records were screened and 42 papers were assessed for eligibility through filtering based on the article titles, abstracts and contents. Endnote software for Windows, version X7 (Thomson Scientific, Stanford, Connecticut, USA) was used to manage the literatures. A narrative review was used in this paper (Zhang et al., 2021). The flow chart of searching literature and identifying the items of self‐management scale for Chinese pregnant women with GDM was shown in Figure 1.

A research team was formed by 7 clinical medical experts including doctors and nurses from the department of obstetrics and endocrinology in the affiliated hospital of Qingdao University. A semi‐structured interview was conducted among the research team members. Based on the literature searching and interview results, an initial self‐management scale for pregnant women with GDM was determined, mainly including self‐management, pregnancy management, blood glucose management, family and social support, with a total of 38 items.

#### 3.1.2 Expert consultation based on Delphi survey

A Delphi survey was conducted to reach consensus on proposed items based on the opinions of the experts. Two rounds of Delphi survey questionnaires were conducted to collect the opinions of experts and to establish final self‐management scale based on expert evaluation and discussion. Twenty‐eight experts were selected from obstetrics department and endocrinology department from 20 hospitals in China. Inclusion criteria for experts: Bachelor degree or above; with an associate professor or professor title; 10 years' work experience in obstetrics or endocrinology department; with the experience of independently treating pregnant women with GDM; volunteer to participate in this study.

Expert consultation form, that is questionnaire, was composed by three parts, survey introduction, each item scoring table and experts’ personal information. The introduction mainly describes the research purpose and background. The importance of items was valued using the Likert five‐point scale. An item with 4–5 point means the expert agree with the item. The inclusion criteria for items: 80% experts agree with the item (importance score ≥ 4 points); average score for item importance > 3.5; variation coefficient < .20. The items can be added, removed or modified based on the expert' opinions. The experts' personal data include age, gender, position and title.

The Delphi survey was conducted by a two‐round questionnaire enquiry. The questionnaire for the first round was formed based the literature search. After the first round of enquiry by email or social software of wechat, the research group added or deleted some items of the scale based on the opinions of the experts. After the second‐round survey, the final self‐management scale was formed with a total of 35 items and was used to test the reliability and validity by the pregnant women with GDM.

### 3.2 Participants

A total of 190 participants were included in the study. Participants were recruited from pregnant women with GDM who seek for antenatal clinic service or were hospitalized in obstetrics department between June and December 2020 in the affiliated hospital of Qingdao University. The participants were explained the purpose of the study and were provided the inclusion and exclusion criteria. Inclusion criteria for pregnant women: (1) meet GDM diagnostic criteria, *Diagnosis Guidelines for gestational diabetes mellitus,* issued in 2004. That is, oral glucose tolerance test was performed for pregnant women after taking 75 g glucose. The blood glucose level was monitored at the moment of empty stomach, 1 hr after taking glucose, or 2 hr after taking glucose. The pregnant women can be diagnosed as GMD if the blood glucose level reaches or exceeds 5.1, 10.0, and 8.5 mmol/L, respectively. It is important to point out that if one of the three items reaches the standard, GMD can be diagnosed; (2) participants with the normal communication and understanding ability and can understand the research and communicate with the research group members freely; (3) be informed the purpose of the research and volunteer to participate in this study. Exclusion criteria: (1) pregnant women with chronic diabetes mellitus complication; (2) with serious diseases, such as severe heart failure and high blood pressure; (3) consciousness disorder, and unable to complete the investigation independently.

### 3.3 Data analysis

The data were processed and analyzed by IBM SPSS Statistics for Windows, version 19 (IBM Corp., Armonk, N.Y., USA). Descriptive analysis is expressed by mean, standard deviation and coefficient of variation. The coefficient of variation is used to represent the dispersion degree of expert opinions. The questionnaire recovery rate is used to show the enthusiasm of experts. The degree of expert authority is represented by the expert authority coefficient. The degree of coordination of expert opinions is reflected by the coefficient of variation and the coefficient of coordination. The smaller value of coefficient of variation means the better coordination of expert's opinions. All the items were scored using a 5‐point Likert‐type scoring method. A standard score was used for the items in the scale. A standard sore is equal to raw score/ theoretical maximum score (Schmitt et al., 2013).

#### 3.3.1 Content validity

The content validity of the items was evaluated by 7 experts. Reliability is a key facet of measurement quality, and split‐half reliability is a statistical method used to measure the consistency of the scores of a test, which is a convenient alternative to other forms of reliability, including test–retest reliability. Test–retest reliability measures repeatability, that is, the degree to which test results are consistent overtime; (Mccrae et al., 2011; Vas et al., 2013). The content validity index (CVI) refers to the ratio of items graded as very or quite relevant by all of the raters involved. The acceptable CVI of items was equal or more than 0.8 (Denise, 2012). Principal component analysis (PCA) was performed to investigate construct validity. Kaiser‐Meyer‐Olkin measure of sampling adequacy (KMO) and Bartlett's test were calculated to evaluate the sample size adequacy. A KMO > 0.80 indicates that the sampling is adequate. The *p* value of Bartlett's test of sphericity should be significant if it is lower than .05 (Liu et al., 2021).

#### 3.3.2 Structural validity

The structure validity of the scale can be evaluated through exploratory factor analysis (EFA). The Cronbach's α coefficient was used to measure the internal consistency reliability (Conway et al., 2021).

## 4 RESULTS

### 4.1 Participants' demographic characteristics

In this study, a total of 190 questionnaires were distributed and 178 were recovered with effective recovery of 93.68%. The age of the participant was 20–38 (30.50 ± 4.50) years; One hundred and fifty‐five (87.08%) women have an education of college or above. One hundred and thirty‐three (74.72%) women are for first birth. One hundred and forty‐eight (83.15%) women are urban residents. One hundred and twenty (67.42%) women use insulin. The detailed demographic characteristics of the participants are presented in Table 1.

**TABLE 1:** Demographic characteristics of participants (*n* = 178)

Characteristics	*n* (%) or mean ± *SD*
Age (years) (mean ± *SD*)	20–38 (30.50 ± 4.50)
Level of education	
High school	23 (12.92%)
College	105 (59.00%)
Post‐graduate	50 (28.08%)
Occupational status	
Employed	135 (75.84%)
Unemployed	43 (24.16%)
Family outcomes each year (RMB)	
>200,000	36 (20.22%)
100,000–200,000	62 (34.83%)
<100,000	80 (44.95%)
Living place	
Urban residents	148 (83.15%)
Countryside residents.	30 (16.85%)
Already has child(ren)	
Yes	45 (25.18%)
No	133 (74.72%)
Insulin use	
Yes	120 (67.42%)
No	58 (32.58%)

### 4.2 Authority and coordination of experts in Delphi survey

Two rounds of expert questionnaire survey were completed. In the first round, twenty‐eight questionnaires were distributed and 26 valid questionnaires were collected with an effective recovery rate of 92.90%. In the second round, twenty‐six copies of questionnaire were distributed, and 25 copies were collected with an effective recovery rate of 96.20%. The authority coefficient of two rounds is 0.88 and 0.91, respectively, which indicates that the experts involved in this study have high authority. In the two‐round survey, the Kendall's W harmony index was 0.12 and 0.11, respectively. It indicates that all experts have a high opinion consistency on the items. Based on the experts' opinion, 3 items were deleted and 4 items were modified. The final self‐management scale includes 4 dimensions and 35 items.

### 4.3 Validity test of self‐management scale

#### 4.3.1 Content validity

The content validity index (CVI) of each item is higher than 0.80, the content validity of each dimension is in range of 0.88–0.96, and the scale content validity is 0.94. All the content validity (including item, dimension and scale) met the criteria, indicating the scale with satisfied content validity (Polit et al., 2007).

#### 4.3.2 Structural validity

The χ2 value of Bartlett's spherical test was 4 352.36 (*p* < .001) and the Kaiser‐Meyer‐Olkin (KMO) value was 0.91, indicating that factor analysis was suitable for this study. Principal component analysis (PCA) and variance maximizing orthogonal rotation were used in the two‐round survey. The first round of exploratory factor analysis (EFA) showed that there were 6 common factors whose eigenvalues > 1 and cumulative variance contribution rate was 61.45%. It is found that the slope gradually getting smaller in the scree graph after the fourth common factor. Therefore, four common factors were extracted for further analysis. In the second round of exploratory factor analysis, the cumulative variance contribution rate of the four common factors was 57.68%, and the factor loadings of each item on the corresponding factor was >0.40. The factor loadings matrix is shown in Table 2. According to the results of factor analysis, the scale structure was adjusted and the factors were named. The scale structure was basically consistent with the original idea. Thus, a final scale for self‐management evaluation of gestational diabetes mellitus was formed, including four dimensions of self‐management awareness, pregnancy management, blood glucose management, and resource utilization, with a total of 35 items.

**TABLE 2:** Factor loading of Self‐management Scale for Chinese pregnant woman with GDM

Items	Factor loading			
	Blood glucose management	Self‐management consciousness	Pregnancy management	Resource utilization
1. I know what foods are good for my blood glucose control	**0.82**	0.16	0.17	0.04
2. I know what foods are bad for my blood glucose control	**0.77**	0.17	0.28	0.04
3. I know how to deal with high blood glucose (adjust diet, exercise, drinking water)	**0.72**	0.23	0.14	0.26
4. I manage my blood glucose on a strict diet	**0.66**	0.02	0.34	0.17
5. I know what to do when I have low blood glucose.	**0.65**	0.19	0.19	0.32
6. I know what the goal of blood glucose control is during pregnancy	**0.63**	0.35	0.03	0.17
7. I do things to prevent hypoglycemia when I exercise (prepare cookies and other foods)	**0.61**	0.03	0.18	0.24
8. I record my blood glucose levels regularly	**0.60**	0.05	0.16	0.18
9. I do regular physical activity to achieve optimal blood sugar levels.	**0.59**	0.04	0.15	0.08
10. My blood glucose is within the standard range during pregnancy	**0.58**	0.15	0.05	0.38
11. When I don't feel well, I will check my blood glucose in time	**0.55**	0.05	0.13	0.43
12. I will monitor my blood glucose regularly	**0.54**	0.26	0.154	0.40
13. I take my weight gain seriously and weigh myself every 1 to 2 weeks	**0.52**	0.14	0.282	0.20
14. My family has enough financial ability to afford the cost of blood glucose management	**0.46**	0.24	0.21	0.35
15. Occasionally I eat lots of sweets or other foods rich in carbohydrates	**0.45**	0.05	0.15	0.09
16. I know that good blood glucose management can reduce my own risk	0.12	**0.81**	0.19	0.06
17. I know that gestational diabetes has bad effects on the fetus	0.14	**0.80**	0.06	0.09
18. I know that gestational diabetes is bad for me	0.12	**0.79**	0.04	0.13
19. I know that good blood glucose management can reduce fetal risk	0.13	**0.79**	0.27	0.02
20. I consider myself directly responsible for blood glucose control	0.08	**0.74**	0.13	0.12
21. I strongly wish I could control my blood glucose well	0.17	**0.67**	0.39	0.03
22. I will actively get the knowledge and management method of GDM through a doctor, a book, the Internet or an app	0.24	**0.56**	0.49	0.08
23. I know the diagnostic criteria for gestational diabetes	0.20	**0.55**	0.13	0.14
24. I will go to see a doctor in time if there are physical discomfort during pregnancy such as abdominal pain, vaginal bleeding, dizziness, headache, fever, and so on	0.09	0.28	**0.60**	0.09
25. I will do 20–30 min of light exercise in 30 min after meals during pregnancy	0.34	0.11	**0.59**	0.05
26. I try to be cheerful during pregnancy	0.15	0.16	**0.58**	0.22
27. I will take calcium, folic acid and iron supplements strictly as prescribed by the doctor during pregnancy	0.11	0.11	**0.57**	0.20
28. I will actively avoid dangerous, smoking, crowded and other bad environment during pregnancy	0.09	0.28	**0.56**	0.02
29. The weight gain of the fetus during the examination was within the normal range	0.26	0.03	**0.55**	0.05
30. I follow the doctor's advice and take prenatal examination on time	0.16	0.31	**0.51**	0.15
311. I will seek help from community health services to manage my blood glucose	0.21	0.02	0.04	**0.76**
32. I will take the initiative to seek a better medical unit to help me manage my blood glucose	0.25	0.06	0.15	**0.75**
33. I will communicate with other pregnant women with GDM about blood glucose management	0.33	0.08	0.15	**0.67**
34. I will ask for help from my family members to manage my blood glucose	0.34	0.158	0.31	**0.54**
35. When blood glucose is not well controlled, I will seek medical advice immediately	0.35	0.06	0.38	**0.46**
Eigen value	12.03	3.52	1.68	1.26
Cumulative Variance Contribution Rate (%)	37.53	10.98	5.24	3.93

Note

The factor loading with bold means that the item mainly belongs to this dimension (one of the four dimensions).

### 4.4 Reliability test of self‐management scale

The Cronbach's α coefficient of the overall scale was 0.95, and the Cronbach's α coefficient of each dimension was 0.78–0.92. The split‐half reliability of the overall scale is 0.79, and the split‐half reliability of each dimension is 0.73–0.90. After 2 weeks, the test–retest reliability of the overall scale was 0.91, and the retest‐reliability of each dimension was 0.76–0.92, both of which were statistically significant difference (*p* < .01) (shown in Table 3).

**TABLE 3:** Reliability of self‐management scale for Chinese pregnant woman with GDM

Dimension	Cronbach's α	Split‐half	Test‐retest
Self‐management consciousness	0.90	0.82	0.85
Pregnancy management	0.78	0.73	0.76
Blood glucose management	0.92	0.90	0.92
Resource utilization	0.82	0.75	0.85
Overall scale	0.95	0.79	0.91

### 4.5 Scoring method for items in self‐management scale

This research used various methods, such as qualitative interview, expert consultation, and reliability and validity test. The formal GDM self‐management scale for pregnant women included four dimensions, self‐management awareness, pregnancy management, blood glucose management, and resource utilization, with a total of 35 items. Likert 5‐level scoring method was used for all items. The items were graded by a standard score, with standard score = (actual score/highest possible score) × 100. It was defined that 60 was poor self‐management ability, 60 ~ 80 was moderate self‐management ability, 80 or higher was good self‐management ability.

## 5 DISCUSSION

The objective of this study was to develop a self‐management scale for pregnant women with GDM and valuate the viability and reliability of the scale in China. Those methods of literature review, qualitative interviews, expert consultation, reliability and validity test were used to develop the formal self‐management scale. The scale includes self‐management awareness, pregnancy management, blood glucose management, and resource utilization, with a total of 35 items.

The participants showed that the self‐management scale possess good validity. Validity includes content validity, structure validity and criterion‐related validity. It is generally believed that a tool can be considered to have good content validity when the content validity of each item is 0.78, and content validity of total scale is 0.90 (Conway et al., 2021; Polit et al., 2007). In this study, the content validity of each item ranged from 0.75 to 1.00, and the content validity of the total scale was 0.94. The results indicated that the content validity of the scale were high. Because no high‐quality self‐management scale for pregnant women with GDM has been found, criterion‐related validity has not been conducted in this study. In exploratory factor analysis, in general, if the common factors extracted from the scale can explain more than 50% of the variation, and each item loaded on the corresponding factor > 0.40, it is considered that the scale has good structural validity. In this study, 4 common factors were extracted, and the cumulative variance contribution rate was 57.68%. The loads of each item on the corresponding factors > 0.40. The final dimension and item attribution are basically consistent with the theoretical hypothesis, which proves that the scale has good structural validity. In subsequent studies, the contents of the items should be discussed and adjusted, while the sample size should be expanded and the sample representativeness should be increased for further testing.

This scale also showed good reliability. Reliability includes stability and internal consistency of the scale. The internal consistency are measured by Cronbach's α coefficient and split‐half reliability. It is generally believed that if the Cronbach's α coefficient of is 0.70–0.80, the internal consistency is acceptable, 0.80–0.90 high; >0.90 very high internal consistency. The split‐half reliability of 0.70 is generally considered as an acceptable standard. The test–retest reliability represents the stability of the scale. The closer the retest reliability value approaches to 1, the higher the stability is. When the test–retest reliability is 0.70, it indicates that the scale has good stability and consistency (Okuroglu et al., 2019). In this study, for the total scale, the Cronbach's α coefficient was 0.95, the split‐half reliability was 0.79, and the test–retest reliability was 0.91. For each dimension, the Cronbach's α coefficient ranged from 0.78 to 0.92, split‐half reliability ranged from 0.73 to 0.90, and the test‐retest reliability ranged from 0.76 to 0.92. The results indicated that the scale had good reliability.

The scale has excellent scientificity and applicability. The development of self‐management scales for patients with general diabetes is relatively mature in China. However, there still exist some limitations during the application of such tools. First, those scales were directly translated from classical foreign scales, such as the Summary of Diabetes Self‐Care Activities Measure (SDSCA; Toobert et al., 2000), which has not experienced cultural adjust and reliability and validity test. Therefore, the reliability and validity are uncertain. Second, most of the self‐made questionnaires have not gone through the standard scale construction process, which cannot ensure scientific and objective. Third, the applicability of some of the items included in the general scale is limited for pregnant women with GDM, such as oral hypoglycemic drugs, smoking, and foot care, which are suit for general chronic diabetes. Last, pregnant women with GDM need good management strategies in blood glucose control, and their pregnancy status should not be ignored. The direct application of self‐management evaluation tools for chronic diabetes is bound to omit the evaluation of pregnancy management. This study combines the characteristics of pregnant women with GDM, gives consideration to the two key contents of blood glucose management and pregnancy management. It emphasizes the self‐management awareness of pregnant women and the reasonable use of external resources. The scale provides multi‐dimensional and more disciplines for the self‐management level of pregnant women with GDM. It is useful to provide basis and evidence for follow‐up intervention research and clinical health education.

It is very interesting and relevant due to the importance of the topic and the implications for the health of pregnant women. Therefore, it is necessary to discuss the relevance or contributions for nursing or health professionals by the fact that women perform self‐management actions. The self‐management scale can provide the pregnant women with GDM more effective diabetes education to ensure clear understanding of self‐management principles. It can assist pregnant women with GDM to better self‐manage their condition and to plan appropriate interventions that can be effective in improving glycaemic control and delaying or preventing diabetes‐related complications for both mother and child, which in turn would decrease the costs of managing the disease. It also provides guidance for nurses–midwives on maternal and postpartum follow‐up care for women at risk or diagnosed with gestational diabetes mellitus in clinical practice.

### 5.1 Limitations

There are some limitations in this study. First of all, this study was based on samples from one hospital, and hence does not represent all Chinese women with GDM. Especially, it cannot represent the pregnant women with GMD from a rural area. The majority of participants in this study are highly educated, it may not be applicable to Chinese women with lower education levels. Besides, most of the experts are selected from level A hospitals in developed cities, whose opinions may be different from those of experts from small towns or rural areas. To overcome the limitations, we recommend to conduct further systematic research at multiple sites among more diverse samples with diverse pregnant women and professional experts. Furthermore, the results of the study cannot be generalized to be outpatients with GDM.

## 6 CONCLUSIONS

A self‐management scale for Chinese pregnant women with GDM was developed in this study. The scale contains 4 dimensions, including self‐management awareness, pregnancy management, blood glucose management, and resource utilization, with a total of 35 items. In the scale, the Cronbach's α was 0.95. The split‐half reliability of the overall scale is 0.79. And the test‐retest reliability was 0.91. The content validity was 0.94. The results showed that the scale had good properties, consistency and validity. It has good reliability and validity and can be used as a tool to evaluate the self‐management level in pregnant women with GDM. However, due to region and time limitations, the sample size and representativeness of this study are limited. Subsequent studies should further increase the sample size, improve the representativeness of samples, and validate and optimize the scale using confirmatory factor analysis.

## CONFLICT OF INTEREST

No conflict of interest has been declared by the authors.

## ETHICAL CONSIDERATION

The study protocol was approved by the Ethics Committee of the affiliated hospital of Qingdao University. The institutional review board has approved the study and waived the need for individual informed consent by formulating a declaration of no objection.

## AUTHOR CONTRIBUTIONS

GK, XM, YW, RX, MZ: Made substantial contributions to conception and design, or acquisition of data, or analysis and interpretation of data. GK, XM, YW, RX, MZ: Involved in drafting the manuscript or revising it critically for important intellectual content. GK, XM, YW, RX, MZ: Given final approval of the version to be published. Each author should have participated sufficiently in the work to take public responsibility for appropriate portions of the content. GK, XM, YW, RX, MZ: Agreed to be accountable for all aspects of the work in ensuring that questions related to the accuracy or integrity of any part of the work are appropriately investigated and resolved.

## Data Availability

The authors confirm that the data supporting the findings of this study are available within the article.
